# The type of the functional cardiovascular response to upright posture is associated with arterial stiffness: a cross-sectional study in 470 volunteers

**DOI:** 10.1186/s12872-016-0281-8

**Published:** 2016-05-23

**Authors:** Anna M. Tahvanainen, Antti J. Tikkakoski, Jenni K. Koskela, Klaus Nordhausen, Jani M. Viitala, Miia H. Leskinen, Mika A. P. Kähönen, Tiit Kööbi, Marko T. Uitto, Jari Viik, Jukka T. Mustonen, Ilkka H. Pörsti

**Affiliations:** School of Medicine, University of Tampere, Tampere, Finland; Department of Mathematics and Statistics, University of Turku, Turku, Finland; Department of Clinical Physiology, Tampere University Hospital, P.O. Box 2000, Tampere, 33521 Finland; Department of Electronics and Communication Engineering, Tampere University of Technology, BioMediTech, Tampere, Finland; Department of Internal Medicine, Tampere University Hospital, P.O. Box 2000, Tampere, 33521 Finland; School of Medicine / Internal Medicine, FIN-33014 University of Tampere, Tampere, Finland

**Keywords:** Arterial stiffness, Cardiac output, Heart rate, Head-up tilt, Systemic vascular resistance

## Abstract

**Background:**

In a cross-sectional study we examined whether the haemodynamic response to upright posture could be divided into different functional phenotypes, and whether the observed phenotypes were associated with known determinants of cardiovascular risk.

**Methods:**

Volunteers (*n* = 470) without medication with cardiovascular effects were examined using radial pulse wave analysis, whole-body impedance cardiography, and heart rate variability analysis. Based on the passive head-up tilt induced changes in systemic vascular resistance and cardiac output, the principal determinants of blood pressure, a cluster analysis was performed.

**Results:**

The haemodynamic response could be clustered into 3 categories: upright increase in vascular resistance and decrease in cardiac output were greatest in the first (+45 % and -27 %, respectively), smallest in the second (+2 % and -2 %, respectively), and intermediate (+22 % and -13 %, respectively) in the third group. These groups were named as ‘constrictor’ (*n* = 109), ‘sustainer’ (*n* = 222), and ‘intermediate’ (*n* = 139) phenotypes, respectively. The sustainers were characterized by male predominance, higher body mass index, blood pressure, and also by higher pulse wave velocity, an index of large arterial stiffness, than the other groups (*p* < 0.01 for all). Heart rate variability analysis showed higher supine and upright low frequency/high frequency (LF/HF) ratio in the sustainers than constrictors, indicating increased sympathovagal balance. Upright LF/HF ratio was also higher in the sustainer than intermediate group. In multivariate analysis, independent explanatory factors for higher pulse wave velocity were the sustainer (*p* < 0.022) and intermediate phenotypes (*p* < 0.046), age (*p* < 0.001), body mass index (*p* < 0.001), and hypertension (*p* < 0.001).

**Conclusions:**

The response to upright posture could be clustered to 3 functional phenotypes. The sustainer phenotype, with smallest upright decrease in cardiac output and highest sympathovagal balance, was independently associated with increased large arterial stiffness. These results indicate an association of the functional haemodynamic phenotype with an acknowledged marker of cardiovascular risk.

**Trial registration:**

ClinicalTrials.gov NCT01742702

**Electronic supplementary material:**

The online version of this article (doi:10.1186/s12872-016-0281-8) contains supplementary material, which is available to authorized users.

## Background

Elevated blood pressure (BP) and related cardiovascular (CV) complications are leading causes of morbidity and mortality, and early recognition of individuals with increased CV risk is of foremost importance [1]. All new cases of CV disease cannot be predicted using classical risk factors like family history, obesity, smoking, hypertension, diabetes, or dyslipidaemias. Therefore, studies aiming at the discovery of novel risk factors are still needed [2, 3]. Furthermore, also psychosocial factors, like hostility and anger, have been linked with worse cardiovascular outcome [4].

The CV phenotype in clinical practice has mainly been determined by measuring brachial BP and HR, even though the value of repeated single BP measurements in the diagnosis of hypertension has been questioned [5, 6]. The haemodynamic changes causing similar elevations of BP may differ between patients and disorders. For example, systemic vascular resistance is typically elevated in essential hypertension [7], while changes in fluid and electrolyte balance are characteristic causes of elevated BP during chronic kidney disease [8].

The age-related decrease in large arterial compliance is accelerated in various CV disorders [9, 10]. Increased large arterial stiffness is an acknowledged CV risk factor in both general populations and subjects with medical disorders [9, 11]. Increased arterial stiffness also predisposes to exaggerated upright decrease in central BP and orthostatic hypotension [12, 13]. The determination of pulse wave velocity (PWV) is the gold standard when evaluating large arterial stiffness [10].

The assessment of CV status is usually performed at rest, but several studies have shown that haemodynamic reactivity to physical stimuli provides further information about CV risk. Enhanced BP response to cold pressor test, or to 4-min 2-step exercise test, predicted the development of hypertension in Japanese populations [14, 15]. Reduced heart rate (HR) recovery after bicycle ergometer testing predicted mortality in a Finnish study [6]. As the change in body posture from supine to upright induces changes in autonomic tone and arterial resistance, orthostatic challenge can also be regarded as a stress test addressing CV reactivity [16, 17].

In the course of our studies on haemodynamics, we have observed that there are reproducible individual variations in the changes in cardiac output and systemic vascular resistance in response to upright posture [17–19]. The objective of the present study was to examine the hypothesis whether functional differences exist in CV responses to upright posture, and whether these differences are associated with known determinants of cardiovascular risk. The results show that not only age, body mass index (BMI), and the presence of hypertension, but also the phenotype of the haemodynamic response to upright posture is associated with arterial stiffness.

## Methods

### Study subjects

All subjects participated in an ongoing study on haemodynamics (clinical trials registration NCT01742702). An announcement for recruitment was distributed at the Tampere University Hospital, University of Tampere, local occupational health care providers, Varala Sports Institute, and two announcements were published in a newspaper. By the time of the present analysis, 694 subjects had been recruited. The participants were interviewed and examined by a physician. Subjects with diabetes, coronary artery disease, cardiac insufficiency, atherosclerotic vascular disease, cerebrovascular disease, renal disease, or medication influencing CV status were excluded.

### Ethics, consent and permissions

All participants gave written informed consent and the study was approved by the ethics committee of Tampere University Hospital District (study number R06086M). The investigation conforms to the principles outlined in the Declaration of Helsinki.

### Laboratory analyses

Blood and urine samples were obtained after about 12 h of fasting. Plasma sodium, potassium, calcium, glucose, creatinine, triglyceride, and total, high-density and low-density lipoprotein cholesterol concentrations were determined using Cobas Integra 700/800 or Cobas 6000, module c501 (F. Hoffmann-LaRoche Ltd, Basel, Switzerland), and blood cell count by ADVIA 120 or 2120 (Siemens Healthcare GmbH, Erlangen, Germany). Estimated glomerulus filtration rate (eGFR) was calculated using the Modification of Diet in Renal Disease (MDRD) formula [20].

### Pulse wave recording

An automatic tonometric sensor on the left wrist was used to continuously capture radial BP and pulse wave form (Colin BP-508 T, Colin Medical Instruments Corp., USA). The radial BP signal was calibrated every 2–4 min by contralateral brachial BP measurements [12, 17, 21, 22].

### Whole-body impedance cardiography

Changes in body electrical impedance during cardiac cycles were recorded using whole-body impedance cardiography (CircMon^R^, JR Medical Ltd., Tallinn, Estonia) to determine HR, stroke volume, and cardiac output, as previously described [23–25]. Cardiac output and stroke volume were related to estimated body surface area to derive cardiac index (l/min/m^2^) and stroke index (ml/m^2^). Systemic vascular resistance index (SVRI, dyn*s/cm^5^*m^2^) was calculated from tonometric BP signal and cardiac index. In supine position and during head-up tilt, the cardiac output values of CircMon^R^ are in good agreement with values measured using thermodilution [23, 24].

PWV was determined using whole-body impedance cardiography [25, 26]. As the impedance-based method slightly overestimates PWV when compared with Doppler ultrasound method, a validated equation was applied to calculate values that correspond to the ultrasound method (PWV = (PWVimpedance *0.696) + 0.864) [25].

The method for PWV measurement using whole body ICG has been described previously [25, 26]. Briefly, the distal impedance is recorded from popliteal artery at knee joint level, and the active electrode is placed on the lateral side of the knee and the reference electrode on the calf below knee. When the pulse pressure wave enters the aortic arch and the diameter of the aorta changes, the whole-body impedance decreases, and this is measured by the voltage electrodes. To calculate the PWV value, the CircMon software measures the time difference between onset of the decrease in impedance in the whole-body impedance signal and the popliteal artery signal (aortic-popliteal PWV). The aortic-popliteal impedance cardiography measurement of PWV has been validated against aortic-popliteal PWV measurements using Doppler ultrasound [25]. The PWV results obtained using CircMon show good repeatability, and normal values for PWV in 799 individuals (age 25–76 years) have been published [26]. We have also shown that the determination of stroke volume using impedance cardiography versus 3-dimensional echocardiography show good correlation [19]. In addition, we have performed carotid-femoral PWV measurements using applanation tonometry and compared them with ICG measurements of aortic-popliteal PWV, and the correlation between these methods was excellent (*r* = 0.82).

### Frequency domain analysis of heart rate variability (HRV)

HRV analysis was used to assess cardiac autonomic tone. The electrocardiograms were recorded by the CircMon^R^ device at 200 Hz sampling rate, and data analyzed using Matlab software (MathWorks Inc., Natick, Massachusetts, USA). Normal R-R intervals were recognized, and a beat was considered ectopic if the interval differed more than 20 % from the previous values. The artifacts were processed using the cubic spine interpolation method [27]. The following frequency domain variables were calculated using the Fast Fourier Transformation method: i) power in low frequency (LF) range (0.04–0.15 Hz), ii) power in high frequency (HF) range (0.15–0.40 Hz), and iii) LF/HF ratio [28].

### Experimental protocol

Caffeine containing products, smoking and a heavy meal for at least 4 h, and alcohol for at least 24 h prior to the investigation were to be avoided. Data was captured in a temperature-controlled laboratory by research nurses [12, 17, 22]. The left arm with the tonometric sensor was abducted 90 degrees in an arm support, which held it at the level of the heart in supine and upright positions. The recording comprised continuous capture of data during three consecutive 5-min periods (total 15 min): 5 min supine, 5 min of head-up tilt to 60 degrees, 5 min supine. The repeatability and reproducibility of the supine and upright measurements are good [22].

### Classification into phenotypes by means of clustering

**Fig. 1:**
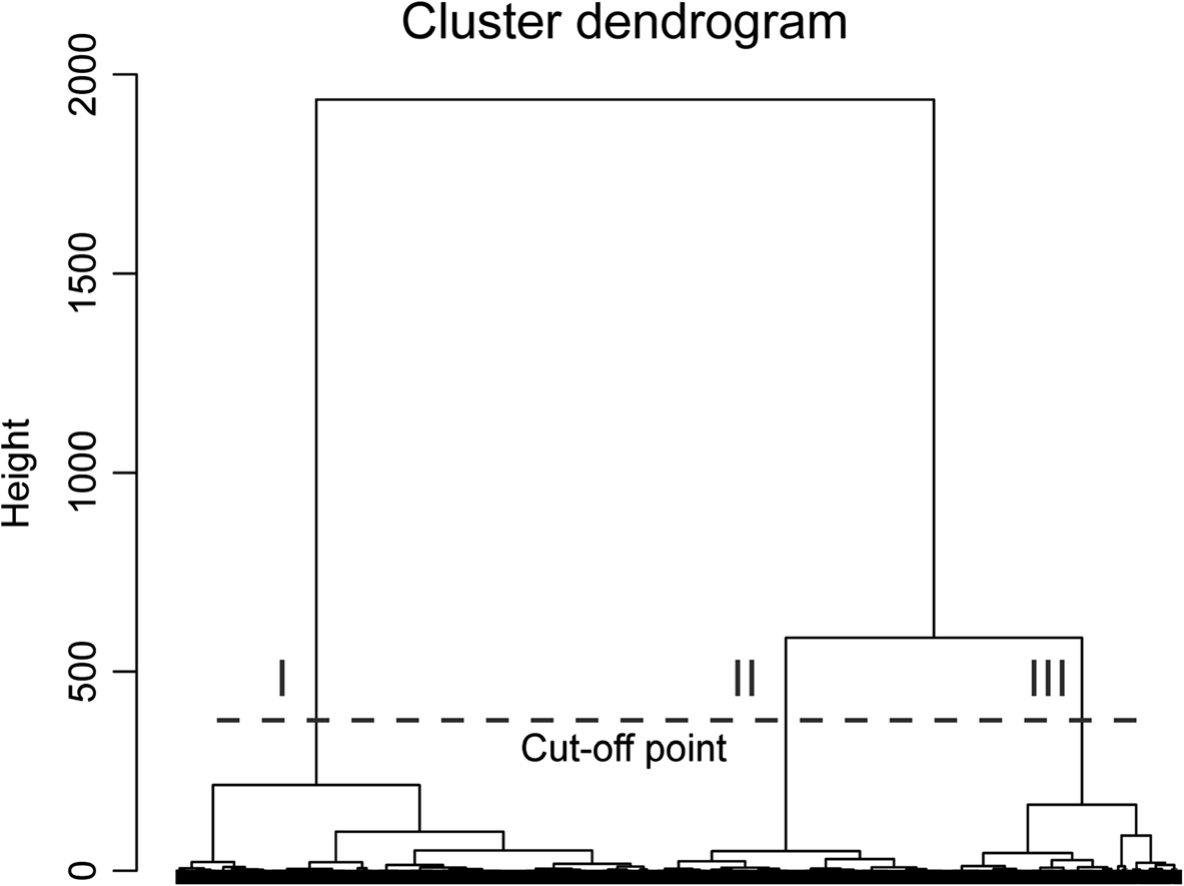
Clustering to phenotypes. Cluster dendrogram according to hierarchical clustering using Ward’s criterion for the squared Euclidean distances. Dashed line indicates the cut-off point for the three phenotypic clusters (I, II and III). The classification was based on the changes in systemic vascular resistance index and cardiac index in response to passive head-up tilt (*n* = 470)

SVRI and cardiac index were chosen for the phenotypic classification, since peripheral arterial resistance and cardiac output are the principal determinants of BP [17, 23, 29]. To characterize the reaction to upright posture, the differences in SVRI and cardiac index between supine values (5th minute average) and upright values (8th and 10th minute averages) were examined. Two values during the head-up tilt (8th and 10th minute) were chosen to take into account possible changes during upright posture. To avoid scaling effects, all differences were standardized to have a mean value of 0 and a variance of 1. Hierarchical clustering was performed using Ward’s criterion for squared Euclidean distances: at first each subject served as a cluster, and at each step two clusters closest to each other were combined, until only one cluster remained (R software version 2.14.1 [30]). Based on the dendrogram, the 3 main clusters were analysed (Fig. 1; classification to clusters I, II and III). We also formed a practical rule for clustering, which was tested in a small validation population (Additional file 1).

### Statistical analyses

Due to skewed distribution, PWV and HRV parameters were logarithmically transformed before statistical analyses. To evaluate the impact of phenotype on PWV, a L1-regression was fitted using sex, categorized BMI, categorized age, categorized hypertension, phenotype, interaction between sex and phenotype (Model 1); and fasting plasma lipids and glucose, and haematocrit (Model 2, with variables of Model 1 included) as variables. In addition, Model 1 was used to evaluate the impact of phenotype on HRV parameters. BMI, age, and presence of hypertension were categorized due to lack of linearity in the data. We performed additional analyses using continuous variables without categorization to examine the association of changes in SVRI and cardiac index with PWV.

There is no accepted cut-off level to define elevated BP during tilt-table measurements, while office BP is usually higher than home BP [5, 31]. As the supine BP during haemodynamic measurements was on average 8/12 mmHg (systolic/diastolic) lower than the seated office BP (*n* = 470), we applied the accepted cut-off level for home and ambulatory daytime measurements, i.e. values ≥135/85 mmHg [31], to define hypertension during the recordings. The same approach was applied previously, with testing of different cut-off levels [17].

As summary statistics, median and 25th to 75th percentile, or mean and standard deviation (SD) or 95 % confidence intervals (CI) were reported. Kruskal-Wallis rank sum test, Fisher’s exact test and Wilcoxon rank sum test were used to compare haemodynamics and demographics between the phenotypes. All analyses were performed using R software version 2.14.1 [30].

## Results

### Study population and average haemodynamic responses to head-up tilt

**Fig. 2:**
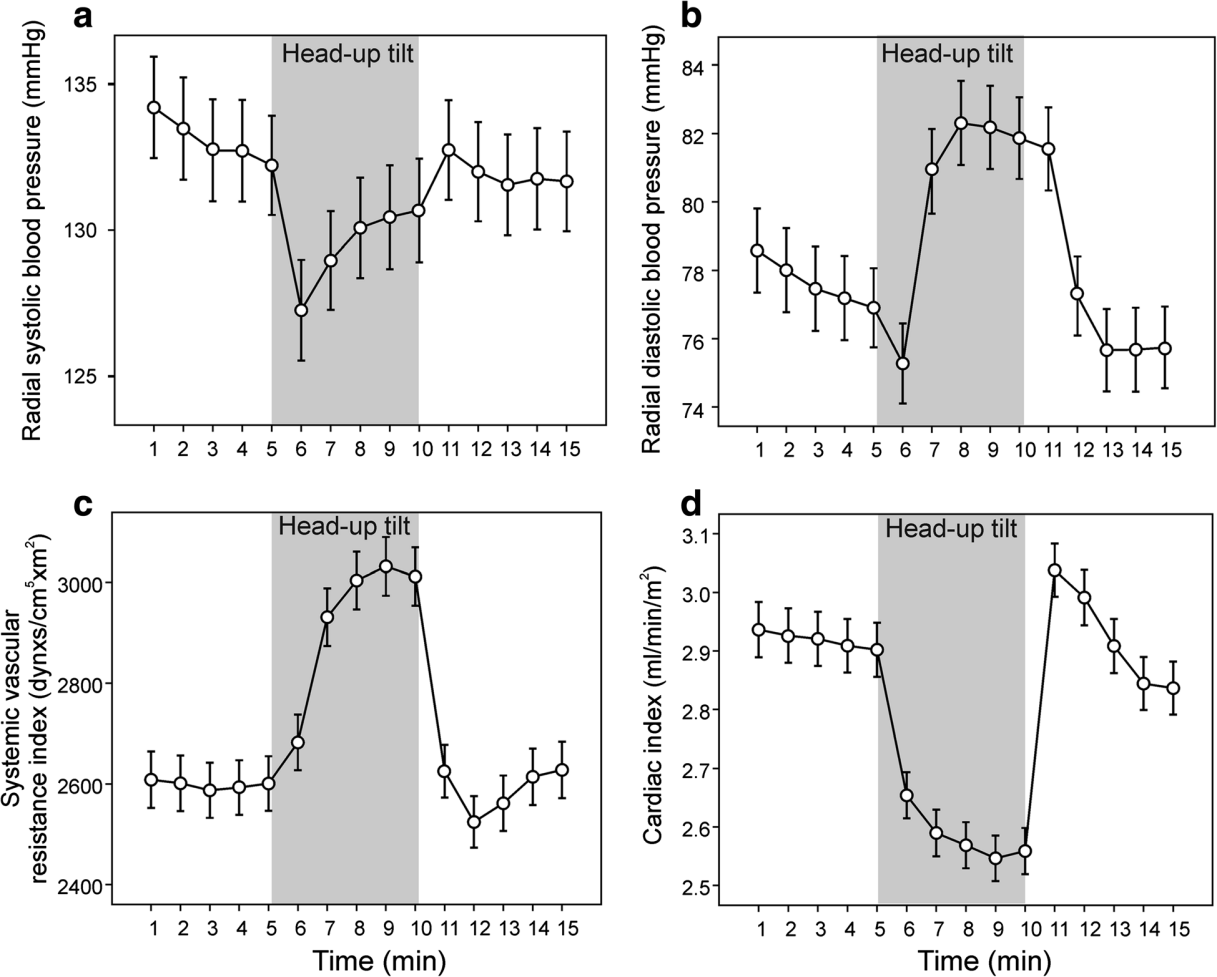
Average haemodynamics during the head-up tilt protocol. Average radial systolic (**a**) and diastolic (**b**) blood pressure, systemic vascular resistance index (**c**), and cardiac index (**d**) during the 15-min measurement protocol (5 min supine – 5 min upright – 5 min supine) in all subjects (*n* = 470). Mean ± 95 % confidence interval

The study population consisted of 470 (240 female and 230 male) individuals aged 20–72 years (average 46 years). Average systolic and diastolic BP, SVRI and cardiac index in all subjects during the 15-min measurement protocol are shown in Fig. 2. In response to upright posture, systolic BP slightly decreased and diastolic BP increased, while SVRI increased by about 15 %, and cardiac index decreased by about 15 %. Both SVRI and cardiac index were stabilized during the time-points chosen for clustering into phenotypes (at 5 min of recording while supine, and at 8 and 10 min of recording while upright) (Fig. 2).

### Classification into phenotypes

**Fig. 3:**
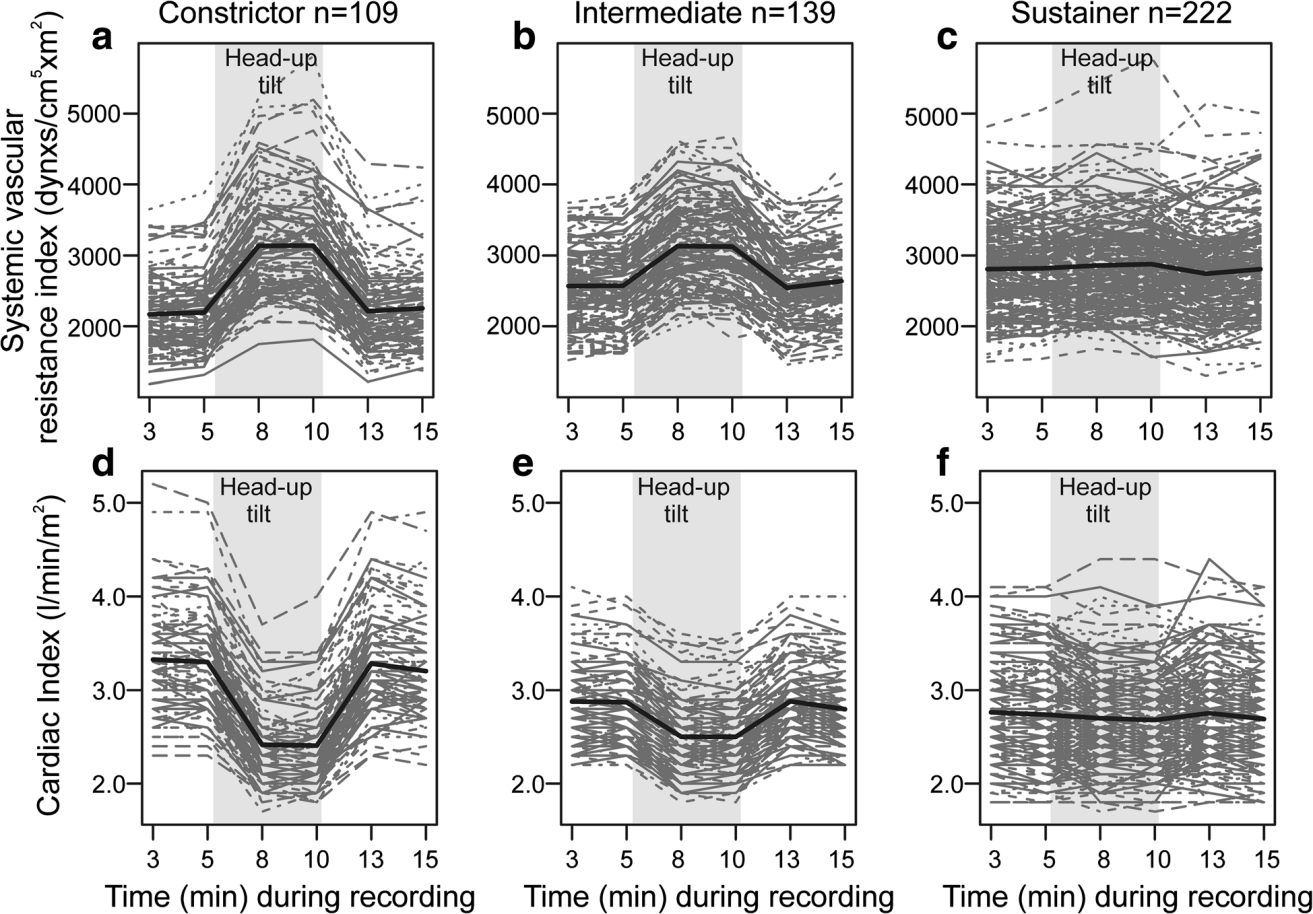
Haemodynamics in the 3 phenotypes in response to upright posture. Systemic vascular resistance index and cardiac index during the measurement protocol, respectively, in the constrictor (**a**, **d**), intermediate (**b**, **e**), and sustainer phenotypes (**c**, **f**). Mean (*bold line*) and individual curves (*grey lines*)

The classification to phenotypes was based on the changes in systemic vascular resistance and cardiac output in response to passive head-up tilt. The cluster that was characterised by the greatest increases in SVRI (+45 %) and the greatest decreases in cardiac index (−27 %) in response to upright posture was named ‘constrictor’ (Fig. 3a, d). The cluster characterised by the smallest changes in SVRI (+2 %) and cardiac index (−2 %), so that these variables were sustained on corresponding levels in supine and upright positions, was named ‘sustainer’ (Fig. 3c, f). The cluster with the intermediary changes (+22 %, -13 %, respectively) was named ‘intermediate’ (Fig. 3b, e). The mean (±SD) head-up tilt -induced changes in SVRI were +944 (±361), +554 (±197) and +60 (±254) dyn*s/cm^5^*m^2^ in the constrictor, intermediate and sustainer phenotypes, respectively (Additional file 2). The mean changes in cardiac index were -0.89 (±0.51), -0.37 (±0.29) and -0.06 (±0.46) l/min/m^2^, respectively (Additional file 2).

Since supine SVRI and cardiac index differed between the clusters, we performed additional analyses in which the subjects were classified to three new clusters merely on the basis of supine levels of SVRI and cardiac index. These new clusters did not systematically or significantly correspond to the constrictor, intermediate and sustainer clusters (data not shown). This indicates that the above phenotypes were not discovered because of differences in the resting levels of SVRI and cardiac index between the groups.

The characteristics and laboratory values of the phenotypes are shown in Table 1. Age did not differ between the groups (*p* = 0.216). The gender distribution (*p* < 0.001), prevalence of laboratory BP ≥135 and/or ≥85 mmHg (*p* = 0.009), BMI (*p* < 0.001), and office BP values (*p* < 0.008) were different in the phenotypes, i.e. the sustainers were more likely to be males with elevated BP and higher BMI than in the other phenotypes (Table 1). In addition, total and LDL cholesterol and triglycerides were lowest, and HDL cholesterol highest in the constrictors (Table 1). Fasting plasma glucose values were lowest in the constrictors, but the mean values were within the normal range in all groups. Haematocrit and creatinine concentrations were lowest in the constrictors, probably due to the female predominance, but the eGFR (MDRD-formula) values did not differ between the phenotypes (Table 1). In analyses adjusted for differences in gender distribution, prevalence of laboratory BP ≥135/85, and BMI, the above differences in total, LDL, and HDL cholesterol, triglycerides, and haematocrit were no more significant between the phenotypes (p-values for all >0.12).

**Table 1:** Demographics and laboratory values in the three phenotypes

Variable	Constrictor	Intermediate	Sustainer	*p*-value
Number of subjects	109	139	222	
Age (years)	46 (37–53)	46 (38–57)	47 (38–55)	0.216
Female (%)	80	58*	32*†	<0.001
BP ≥ 135/85 mmHg (%) in the laboratory	34	42*	51*†	0.009
Body mass index (kg/m^2^)	23.1 (21.6–25.6)	26.5 (24.1–29.3)*	27.4 (24.8–30.6)*	<0.001
Waist circumference (cm)				
Female	79 (73–87)	86 (79–97)*	88 (82–99)*	<0.001
Male	85 (80–92)	98 (91–99)*	100 (94–107)*	<0.001
Smoking (no/present/previous)	66/16/27	90/14/35	121/23/78	0.090
Office systolic BP (mmHg)	134 (119–148)	139 (123–152)	141 (123–157)*	0.007
Office diastolic BP (mmHg)	84 (75–91)	90 (81–98)*	91 (82–99)*	<0.001
Haematocrit	0.40 (0.39–0.42)	0.42 (0.39–0.44)*	0.43 (0.41–0.45)*†	<0.001
Creatinine (μmol/l)	66 (59–74)	70 (64–80)*	76 (69–84)*†	<0.001
eGFR (ml/min/1.73 m^2^)	89 (80–97)	88 (79–97)	89 (78–99)	0.631
Total Cholesterol (mmol/l)	4.80 (4.20–5.50)	5.20 (4.60–5.80)*	5.20 (4.4–5.8)*	0.018
LDL Cholesterol (mmol/l)	2.45 (2.10–3.18)	3.00 (2.40–3.60)*	3.10 (2.30–3.70)*	<0.001
HDL cholesterol (mmol/l	1.78 (1.49–2.13)	1.54 (1.23–1.84)*	1.43 (1.17–1.71)*	<0.001
Triglycerides (mmol/l)	0.92 (0.61–1.16)	1.08 (0.82–1.43)*	1.11 (0.76–1.67)*†	<0.001
Fasting plasma glucose (mmol/l)	5.2 (4.9–5.4)	5.4 (5.1–5.7)*	5.5 (5.1–5.8)*	<0.001

Values are median (25th-75t^h^ percentile); BP, blood pressure; eGFR, estimated creatinine-based glomerulus filtration rate using the MDRD formula;^23^ *p < 0.05 when compared with constrictor phenotype; †*p* < 0.05 when compared with intermediate phenotype

### Detailed haemodynamics and arterial stiffness

The sustainers showed highest supine systolic BP, while upright systolic BP did not differ between the phenotypes (Additional files 3 and 4). Constrictors showed numerically lowest supine and upright diastolic BP. Supine heart rate was lowest and upright heart rate highest in sustainers, while supine stroke index was numerically highest and upright stroke index lowest in the constrictors (Additional file 3). Subsequently, supine cardiac index was highest but upright cardiac index was lowest in the constrictors. Supine SVRI was lowest in the constrictors, while upright SVRI was lowest in the sustainers (Additional file 3, Fig. 3).

**Fig. 4:**
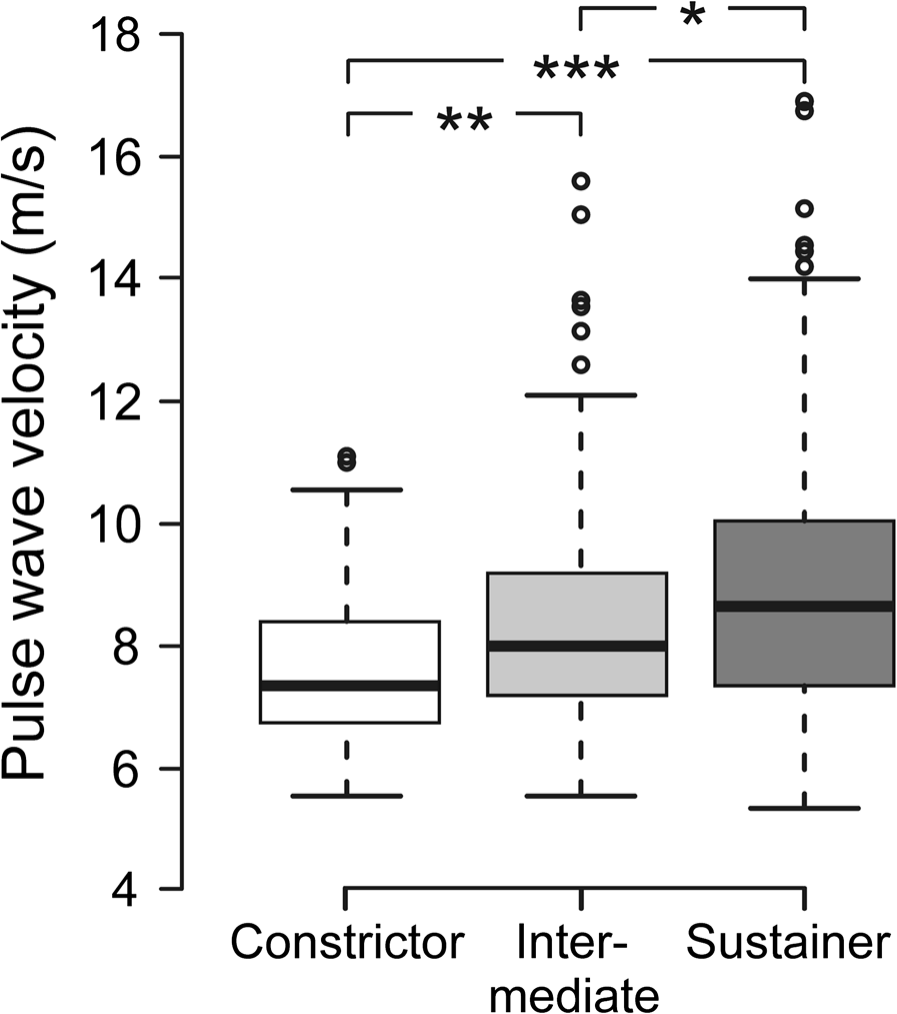
Arterial stiffness in the 3 phenotypes. Boxplots depicting supine pulse wave velocity in the constrictor, intermediate and sustainer phenotypes. Median (*line inside box*), 25th to 75th percentile (*box*), range (+), and outliers (*open circles*); *p < 0.05, **p < 0.01, ***p < 0.001

Median PWV was different between the phenotypes, sustainers showing the highest values (7.35, 8.00 and 8.65 m/s in the constrictor, intermediate and sustainer phenotypes, respectively) (Fig. 4). In multivariate analysis, sustainer and intermediate phenotypes, high and middle BMI tertiles, high and middle age tertiles, BP ≥135/85 mmHg, and interaction between female sex and intermediate phenotype were significant explanatory factors for supine PWV (Table 2, Model 1). If the laboratory variables that were different between the phenotypes (plasma lipids and glucose, haematocrit) were included in the multivariate model, the outcome was that sustainer and intermediate phenotype, high and middle BMI tertiles, high and middle age tertiles, BP ≥135/85 mmHg, interaction between female sex and intermediate phenotype, and plasma triglyceride level were significant explanatory factors for supine PWV (Table 2, Model 2). In additional analyses using continuous variables without categorization, the supine to upright change in cardiac index was still independently associated with PWV, while the association between the change in SVRI and PWV was not significant (Additional file 5).

**Table 2:** Analysis of determinants of arterial stiffness

	Model 1: Constant 1.83258		Model 2: Constant 1.74717	
Variable	Coefficient	*p*-value	Coefficient	*p*-value
Phenotype: intermediate	0.09103	0.046	0.12729	0.005
Phenotype: sustainer	0.09103	0.022	0.11514	0.005
BMI: middle tertile	0.07742	<0.001	0.07209	0.002
BMI: high tertile	0.12824	<0.001	0.11146	<0.001
Age: middle tertile	0.09143	<0.001	0.08398	<0.001
Age: high tertile	0.23804	<0.001	0.23729	<0.001
Blood pressure ≥135/85 mmHg	0.10273	<0.001	0.08734	<0.001
Female sex	0.03609	0.377	0.08017	0.065
Interaction: female sex and intermediate phenotype	−0.11124	0.043	−0.14789	0.007
Interaction: female sex and sustainer phenotype	−0.07442	0.143	−0.09407	0.071
Fasting plasma triglycerides	-	-	0.05707	0.004

Analysis of explanatory factors for supine pulse wave velocity (after logarithmic transformation) in multivariate analysis. The male sex, constrictor phenotype, lowest BMI tertile, lowest age tertile, and BP < 135/85 mmHg served as reference categories in both models, i.e. for these variables the numeric value of the coefficient is 0

Model 1 was based on phenotype, BMI-category, age-category, BP-category, and sex. *Model 1 formula for log(PWV)* ~ Constant + phenotype + BMI-category + AGE-category + BP-category + sex + phenotype*sex

Example of interpretation: median log(PWV) for a woman with the sustainer phenotype with BMI and age in the highest tertile and BP ≥ 135/85 mmHg: 1.83258 + 0.09103 + 0.12824 + 0.23804 + 0.10273+ 0.03609 + (-0.07442) = 2.35429; median PWV = exp(2.35429) = 10.53 m/s

Model 2 was based on Model 1 and fasting plasma total, LDL, and HDL cholesterol, triglycerides, glucose, and haematocrit

*Model 2 formula for log(PWV)* ~ Constant + phenotype + BMI-category + AGE-category + BP-category + sex + phenotype *sex + 0.05707*plasma triglyceride concentration

### Heart rate variability in the phenotypes

**Fig. 5:**
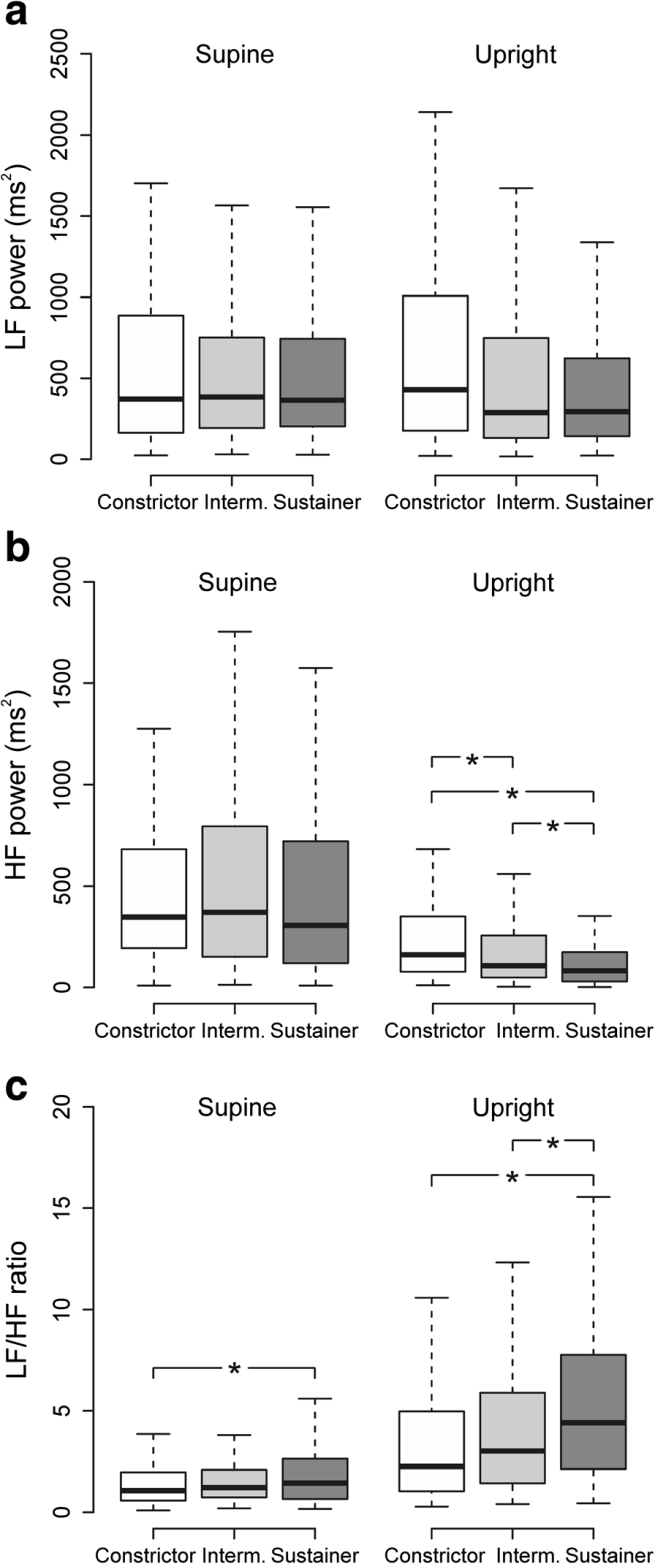
Heart rate variability in the 3 phenotypes. Boxplots depicting low frequency (LF) power (**a**), high frequency (HF) power (**b**), and LF/HF ratio (**c**) of heart rate variability in supine and upright positions. Median (*line inside box*), 25th to 75th percentile (*box*), and range (*whiskers*); *p < 0.05, **p < 0.01, ***p < 0.001

The HRV variables in supine and upright position are depicted in Fig. 5. No significant differences were found between the phenotypes in supine or upright power in LF range, a variable predominantly reflecting cardiac sympathetic tone [28, 32]. Supine power in HF range did not differ between the phenotypes, either. However, upright HF power was highest in the constrictors, followed by the intermediate phenotype, while the sustainer phenotype showed lowest level, suggesting reduced cardiac parasympathetic tone [28, 32]. The sympathovagal balance, as reflected by LF/HF ratio, was higher in supine position in the sustainers than in the constrictors, while upright LF/HF ratio was higher in the sustainers than in the two other phenotypes (Fig. 5). In all phenotypes, LF/HF ratio clearly increased in the upright position (*p* < 0.001). When the analyses were adjusted for differences in sex, BMI, and presence of hypertension, there were no statistically significant differences in the HRV variables between the phenotypes (Additional file 6).

### Medications used by the subjects

The majority (58 %) of subjects were without any medications, and none had medication for cardiovascular disorders or diabetes. The established medications used by the participants were: 75 female subjects on low-dose hormones (oestrogen, progestin or combination) for contraception or hormone replacement therapy, 22 treated with antidepressants, 16 with thyroxin, 21 with intranasal or inhaled corticosteroids, 12 with statins, 11 with proton pump inhibitors, 11 with antihistamines, 5 with non-steroidal anti-inflammatory drugs, and 5 with glucosamine. Female hormone regimens were used by 31 % of the female subjects, but the proportions of low-dose hormone users did not differ between the phenotypes. Only the use of inhaled or intranasal corticosteroids (*p* = 0.038) differed between the groups so that these compounds were used by 6.4 %, 6.5 % and 2.3 % of the subjects in the constrictor, intermediate, and sustainer groups, respectively.

## Discussion

The evaluation of the CV status only in the supine or seated position gives rather limited information about haemodynamics [16, 17, 22]. Although head-up tilt for 5 min is a short period of observation, we found that haemodynamic adaptation differed between individuals of similar age who only showed small differences in BP (Additional files 2 and 3). The type of haemodynamic reaction to upright posture was also related to PWV, an acknowledged measure of large arterial stiffness [9–11]. Previously, the majority of studies utilizing head-up tilt have been performed to examine the mechanisms of syncope [33, 34], and differences in upright haemodynamics have not been systematically investigated in non-syncopal subjects.

Subjects with increased CV risk should be identified before the manifestations of a clinical disease [1]. In addition to the classical risk factors, efforts have been made to identify novel risk factors to increase the sensitivity of risk evaluation. For example, haemodynamic responses to physical challenge (bicycle exercise test, step exercise test, cold pressor test) can predict CV outcome [6, 15, 35]. As change in posture from supine to upright activates sensory and neurogenic responses in the body, with subsequent changes in autonomic tone, cardiac function, and peripheral arterial resistance, passive head-up tilt can be regarded as a clinical haemodynamic stress test [12, 16, 17].

Here we used systemic vascular resistance and cardiac index, the principal determinants of BP [17, 23, 29], in the classification of haemodynamic response to upright posture into three phenotypes. The constrictor phenotype showed highest increase in SVRI, greatest decrease in cardiac output, and highest upright HF power as an indicator of parasympathetic cardiac autonomic tone [28, 32]. The sustainers showed lowest increase in vascular resistance and smallest decrease in cardiac output, and greatest upright LF/HF ratio indicating highest sympathovagal balance [28, 32]. The constrictors were also characterised by a more favourable CV risk profile than the sustainers, with lower total and LDL cholesterol, triglycerides and glucose (Table 1). However, in analyses adjusted for the differences in gender distribution, BMI and BP, the differences in the lipid and glucose values were no more significant.

In multivariate analysis including BMI, age, BP, gender, haematocrit, plasma glucose and lipid profile, the sustainer and intermediate phenotypes were associated with higher PWV, i.e. increased large arterial stiffness, than constrictors. Of note, the statistical coefficients relating the intermediate and sustainer phenotypes to higher PWV corresponded to those of increased BMI and elevated BP (Table 2). Higher PWV has been recognised as an independent CV risk factor in hypertensive subjects, elderly subjects, diabetics, and even in the general population [9, 36, 37]. The close relation between PWV and the well-known CV risk factors, increased BMI and BP, has been repeatedly shown [17, 38, 39]. As ageing is a strong determinant of PWV [37, 40], it is important to note that the average age did not differ between the three phenotypes in the present study.

In supine position, HRV analysis showed a higher LF/HF ratio (increased symphatovagal balance) in the sustainers than in the constrictors [28, 32]. Supine SVRI was also highest in the sustainers, who were characterised by male predominance, and highest BMI and BP, all factors associated with increased sympathetic tone [41–43]. The head-up tilt uncovered differences in cardiac autonomic tone that largely resulted from the suppression of cardiac parasympathetic tone (decrease in HF power) in response to upright posture in the intermediate and sustainer phenotypes (Fig. 4b). Subsequently, the sustainers showed the highest upright LF/HF ratio, which corresponds to the smallest upright decrease in cardiac output in this phenotype. Previously, sympathovagal imbalance in young prehypertensive subjects has been attributed to increased sympathetic and decreased parasympathetic tone [44].

Increased arterial stiffness is known to influence the control of autonomic tone. Higher arterial stiffness has been associated with reduced heart rate variability (HRV) [45–47]. As baroreceptors are located in the arterial wall, arterial stiffening attenuates baroreceptor responses to changes in BP [48]. Increased large arterial stiffness and impaired cardiac baroreflex control are also typical features of hypertension, and haemodynamic changes in hypertensive subjects have been attributed to reduced vagal inhibitory influence and overdrive of the sympathetic nervous system (for a review, see [49]). Yet, reduced baroreflex sensitivity in the elderly can persist during methodological elimination of the influence of the stiffness of the vessel wall [50]. Therefore, increased arterial stiffness is not the sole explanation for baroreflex changes, and these may also result from changes in the control of neural baroreflex pathways [50].

In the present study, a putative explanation for the association between the sustainer phenotype and increased arterial stiffness would be alterations in baroreflexes, and this topic is a subject for further studies. However, it should be noted that the differences in HF power and LF/HF ratio were no more significant in adjusted analyses. This indicates that the demographic differences (sex distribution, BMI, and presence of hypertension) between the phenotypes largely explained the observed deviations in cardiac autonomic tone. Importantly, the differences in PWV between the phenotypes persisted after the adjustments, indicating that the deviations in autonomic tone did not explain the differences in arterial stiffness. The cross-sectional design of our study does not allow conclusions about causality, and an unanswered question is whether modifications in the associating risk profiles (like weight reduction) would result in changes of the functional cardiovascular phenotype.

Gender distribution was different between the phenotypes, with male predominance in the sustainers. Previously, haemodynamic differences between men and women have been observed in central wave reflections [51], but the functional CV differences have been less thoroughly characterized. In women, autonomic responses to orthostasis may be attenuated due to lower baroreceptor sensitivity [52]. Smaller body size and lower centre of gravity have been suggested to increase venous pooling of blood to lower extremities in women during orthostatic challenge [53]. In the present study, the decrease in cardiac index during upright posture was greatest in the constrictors with female predominance, which could imply an increase in venous pooling of blood during head-up tilt. However, BP was well maintained in the constrictors, probably due to the pronounced increase in vascular resistance. Despite the differences in gender distribution between the groups, PWV was significantly associated with the functional phenotype, while the association of PWV with sex was only found as an interaction in the intermediate phenotype.

The present non-invasive methods have been validated against invasive methods [23, 29, 54], and the repeatability and reproducibility of the measurements are good [22]. The non-invasive nature of the recordings is a limitation, as the calculation of cardiac output from the bioimpedance signal requires mathematical equations and simplification of physiology [24]. Invasive haemodynamic measurements, however, are not justified in humans without a clinical reason. The tonometric measurement of carotid-femoral PWV is considered as the gold standard for the assessment of arterial stiffness [55], and the lack of this method in our study can be considered as a limitation. The impedance cardiography-derived PWV shows good correlation with PWV measured with Doppler ultrasound [25], and the method has also been found to be a practical approach for the evaluation of arterial stiffness in 799 individuals aged 25–76 years [26]. The median BMI in the study population was 26.3 kg/m^2^, which corresponds to the average BMI in Finnish men (27.4 kg/m^2^) and women (26.9 kg/m^2^) in a large Finriski 2007 survey [56]. The present median office BP (138/89 mmHg) was slightly lower than the reported average BP in the Finnish population (145-148/85-90 mmHg) [57]. Importantly, subjects taking medications with known influences on haemodynamics were excluded from the present study.

## Conclusions

We found that subjects could be classified to 3 phenotypes according to the head-up tilt –induced changes in CV function. Independently of other risk factors, the sustainer and the intermediate phenotypes were associated with increased large arterial stiffness. Evaluation of cardiac autonomic tone showed increased supine and upright sympathovagal balance in the sustainer phenotype. The present results suggest that the functional phenotype of the haemodynamic response to upright posture is associated with arterial stiffness and thus also with the level of CV risk. Future follow-up studies with CV end-points are needed to demonstrate the clinical relevance of the present phenotypic information.

## Abbreviations

BMI, body mass index; BP, blood pressure; CI, confidence interval; CV, cardiovascular; eGFR, estimated glomerular filtration rate; HDL, high-density lipoprotein; HF, high frequency; HR, heart rate; HRV, heart rate variability; LDL, low-density lipoprotein; LF, low frequency; LF/HF, low frequency/high frequency ratio; MDRD, modification of diet in renal disease; PWV, pulse wave velocity; SD, standard deviation; SVRI, systemic vascular resistance index.

## Additional files

Additional file 1: Figure and Table in pdf-format showing practical classification rule tree based on the head-up tilt –induced changes in systemic vascular resistance index and cardiac index, and contingency table showing the agreement between two clustering procedures (hierarchical clustering and practical rule) in the original data (*n* = 470) and in the validation data (*n* = 30). (PDF 182 kb) Additional file 2: Figure in pdf-format showing the magnitude of the changes in systemic vascular resistance index and cardiac index in response to upright posture in the 3 phenotypes. (PDF 75 kb) Additional file 3: Table in pdf-format containing data about the supine and upright haemodynamics in the three phenotypes. (PDF 80 kb) Additional file 4: Figure in pdf-format showing radial systolic and diastolic blood pressure during the measurement protocol in the 3 phenotypes. (PDF 437 kb) Additional file 5: Table in pdf-format showing the analysis of explanatory factors for supine pulse wave velocity in multivariate analysis using continuous variables without categorization. (PDF 13 kb) Additional file 6: Table in pdf-format showing outcome of adjusted analyses of heart rate variability parameters in supine and upright positions using model 1 presented in Table 2. (PDF 13 kb)
