# Supplementary material for: The type of the functional cardiovascular response to upright posture is associated with arterial stiffness: a cross-sectional study in 470 volunteers
Source: BMC Cardiovasc Disord. 2016 May 23;16:101. doi: 10.1186/s12872-016-0281-8 (PMC4877753; doi:10.1186/s12872-016-0281-8)
Supplement: Additional file 1: — Figure and Table in pdf-format showing practical classification rule tree based on the head-up tilt –induced changes in systemic vascular resistance index and cardiac index, and contingency table showing the agreement between two clustering procedures (hierarchical clustering and practical rule) in the original data (n = 470) and in the validation data (n = 30). (PDF 182 kb) [file 12872_2016_281_MOESM1_ESM.pdf]

Additional file 1. Practical rule for clustering. Classification rule tree as based on the head-up tilt –induced changes in systemic vascular resistance index (SVRI, dyn*s/cm^5*m^2) and cardiac index (CI, ml/min/m^2). T1 stands for supine values (average of the 5th minute of the recording), T2 and T3 for the head-up tilt values (average values of the 8th and 10th minutes of the recording, respectively).

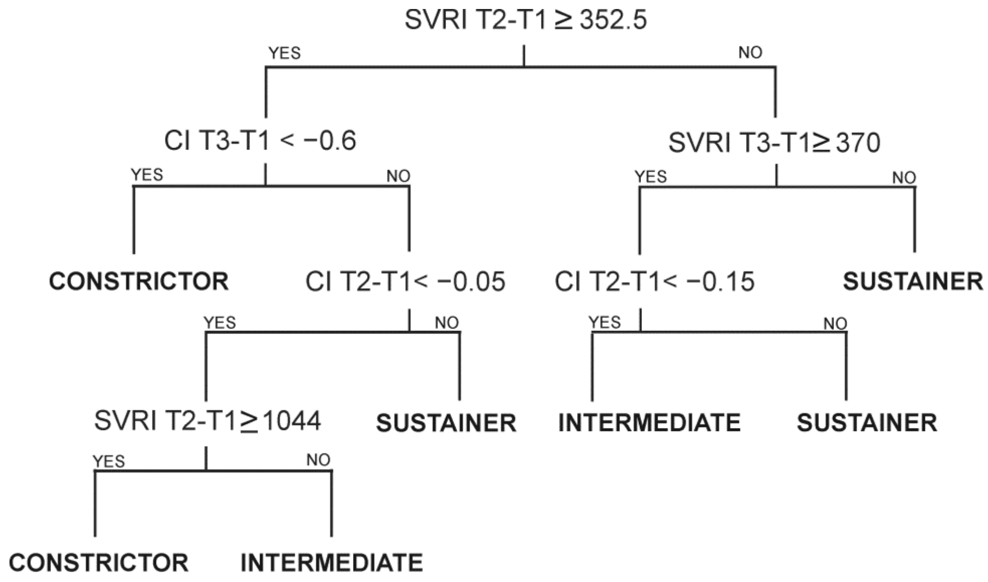

Contingency table showing the agreement between the clustering procedures. Analyses performed with hierarchical clustering (columns) and practical rule (rows) in the original data (A) and in the validation data (B). Numbers in bold font represent subjects who were classified into the same cluster with both methods.

	Hierarchical clustering		
Practical rule	Constrictor	Intermediate	Sustainer
A. Original data (N=470)			
Constrictor	**108**	9	0
Intermediate	1	**126**	4
Sustainer	0	4	**218**
B. Validation data (N=30)			
Constrictor	**6**	1	0
Intermediate	0	**4**	1
Sustainer	0	1	**17**

Practical classification rule and validation

The statistical practical rule for clustering was based on head-up tilt -induced changes in SVRI and cardiac index. When we tested the contingency between the hierarchical clustering above and the classification rule, only 18 (3.8%) of the 470 subjects were misclassified using the practical rule. All misclassified subjects were shifted out from, or into, the intermediate cluster, i.e. no misclassifications between the extreme clusters were observed. In the validation data obtained with additional 30 participants (recorded after the analysis of the results of the above 470 subjects), altogether 3/30 subjects (10%) were misclassified using the rule, and none of the subjects were shifted between the extreme clusters.
