# Supplementary material for: The type of the functional cardiovascular response to upright posture is associated with arterial stiffness: a cross-sectional study in 470 volunteers
Source: BMC Cardiovasc Disord. 2016 May 23;16:101. doi: 10.1186/s12872-016-0281-8 (PMC4877753; doi:10.1186/s12872-016-0281-8)

Additional file 2. **Magnitude of changes in systemic vascular resistance and cardiac index in response to upright posture.** Changes in systemic vascular resistance index (A) and cardiac index (B) from the average of the 5th minute of the recording (supine) to the average of the 8th minute of the recording (upright) in the constrictor, intermediate, and sustainer phenotypes. Bar graphs depict mean and whiskers 95% confidence interval of the mean.

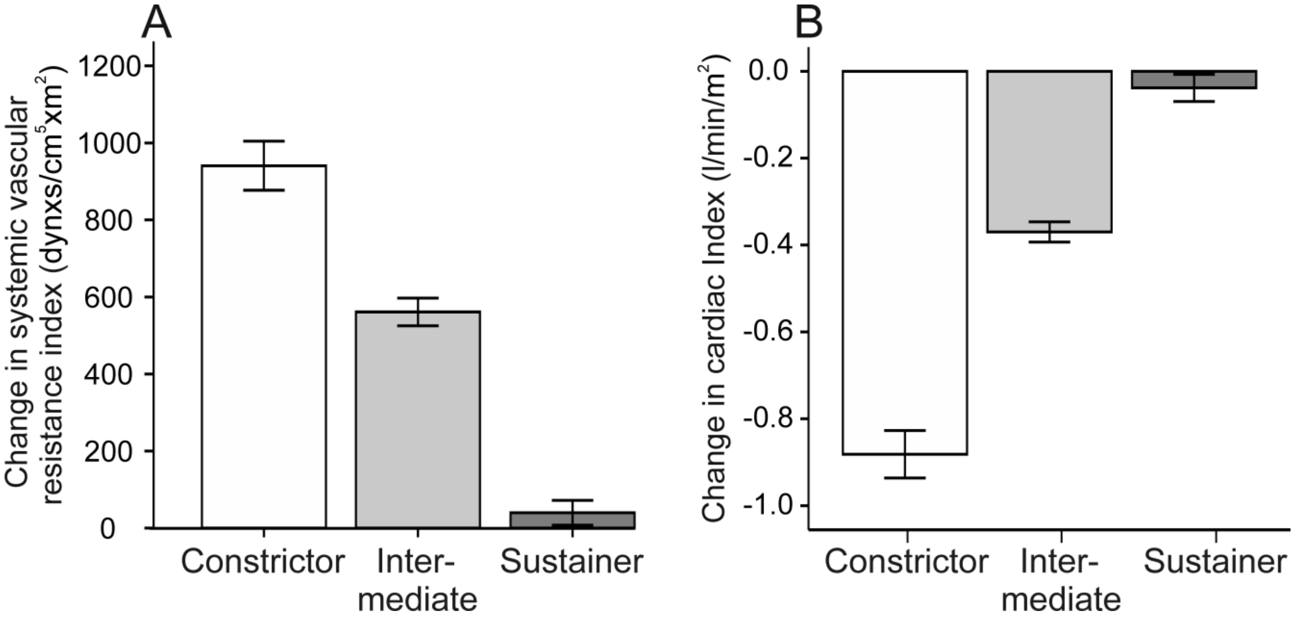

Supplement: Additional file 2: — Figure in pdf-format showing the magnitude of the changes in systemic vascular resistance index and cardiac index in response to upright posture in the 3 phenotypes. (PDF 75 kb) [file 12872_2016_281_MOESM2_ESM.pdf]
