# Supplementary material for: The type of the functional cardiovascular response to upright posture is associated with arterial stiffness: a cross-sectional study in 470 volunteers
Source: BMC Cardiovasc Disord. 2016 May 23;16:101. doi: 10.1186/s12872-016-0281-8 (PMC4877753; doi:10.1186/s12872-016-0281-8)
Supplement: Additional file 3: — Table in pdf-format containing data about the supine and upright haemodynamics in the three phenotypes. (PDF 80 kb) [file 12872_2016_281_MOESM3_ESM.pdf]

Additional File 3. Supine and upright haemodynamics in the three phenotypes.

Variable	Constrictor	Intermediate	Sustainer	p-value
Number of subjects	109	139	222	
Supine radial systolic BP (mmHg)	127 (113-142)	130 (117-144)	135 (123-146)*†	0.004
Upright radial systolic BP (mmHg)	128 (112-144)	130 (119-146)	129 (116-140)	0.202
Supine radial diastolic BP (mmHg)	70 (65-83)	77 (67-84)	79 (71-89)*†	<0.001
Upright radial diastolic BP (mmHg)	78 (68-89)	84 (77-94)*	82 (73-92)	0.017
Supine heart rate	64 (59-69)	62 (57-69)	60 (56-67)*	0.018
Upright heart rate	73 (66-79)	74 (66-82)	76 (71-83)*	0.013
Supine stroke index (ml/min/m2)	50 (47-55)	45 (41-49)*	44 (41-47)*	<0.001
Upright stroke index (ml/min/m2)	32 (30-36)	33 (30-38)	35 (32-38)*†	<0.001
Supine cardiac index (l/min/m^2)	3.25 (3.00-3.60)	2.80 (2.55-3.10)*	2.70 (2.40-3.05)*†	<0.001
Upright cardiac index (l/min/m^2)	2.32 (2.10-2.55)	2.45 (2.28-2.70)*	2.65 (2.35-2.95)*†	<0.001
Supine SVRI (dyn*s/cm^5*m^2)	2104 (1800-2495)	2550 (2152-2920)*	2824 (2451-3125)*†	<0.001
Upright SVRI (dyn*s/cm^5*m^2)	3063 (2577-3538)	3103 (2679-3505)	2808 (2454-3200)*†	<0.001

Values are median (25th to 75th percentile); BP, blood pressure; SVRI, systemic vascular resistance index;

*p<0.05 when compared with constrictor phenotype; †p<0.05 when compared with intermediate phenotype.
