# Supplementary material for: The type of the functional cardiovascular response to upright posture is associated with arterial stiffness: a cross-sectional study in 470 volunteers
Source: BMC Cardiovasc Disord. 2016 May 23;16:101. doi: 10.1186/s12872-016-0281-8 (PMC4877753; doi:10.1186/s12872-016-0281-8)

Additional file 4. **Blood pressure in response to head-up tilt.** Radial systolic and diastolic blood pressure during the measurement protocol, respectively, in the constrictor (A, D), intermediate (B, E), and sustainer phenotypes (C, F). Mean (bold line) and individual curves (grey lines).

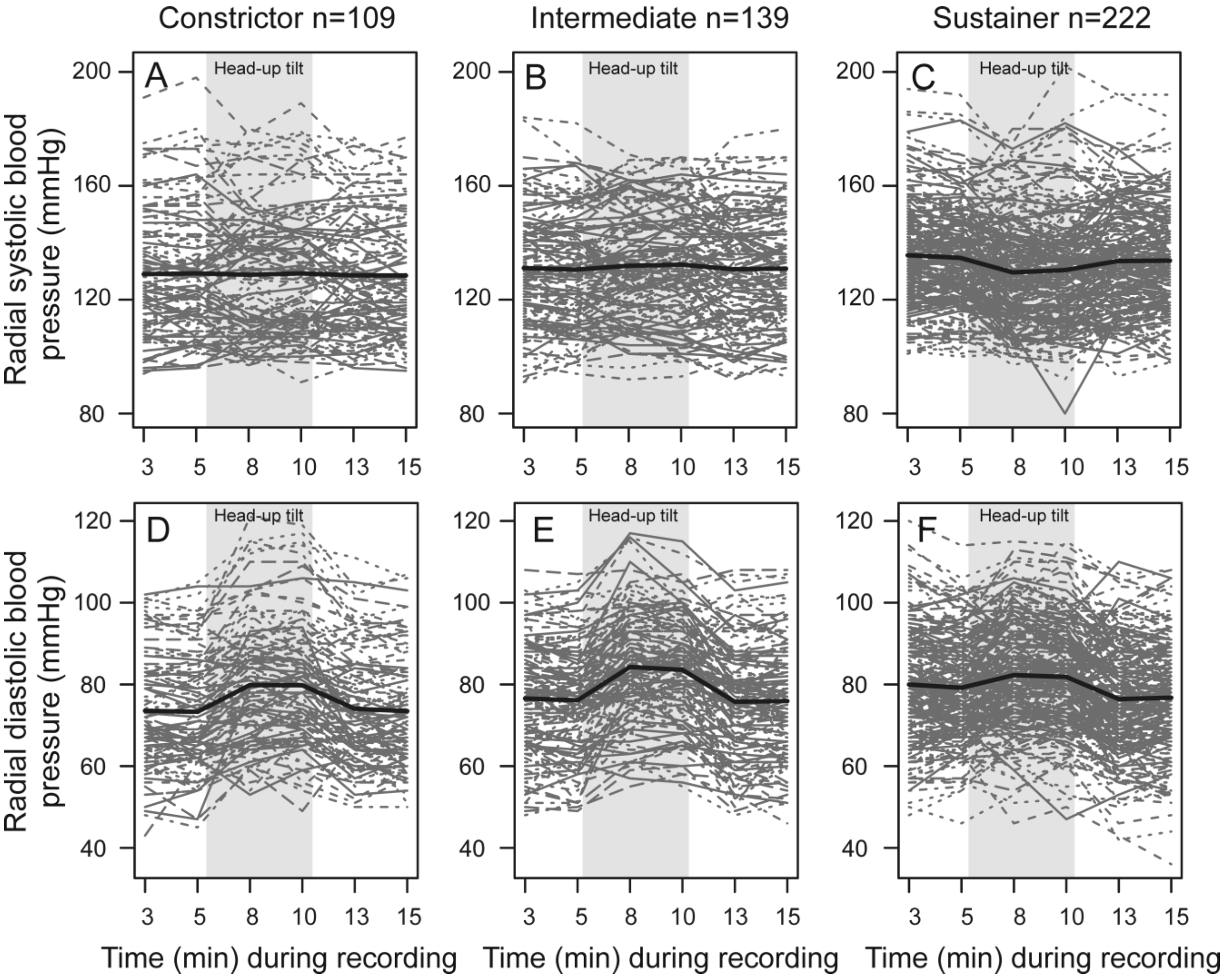

Supplement: Additional file 4: — Figure in pdf-format showing radial systolic and diastolic blood pressure during the measurement protocol in the 3 phenotypes. (PDF 437 kb) [file 12872_2016_281_MOESM4_ESM.pdf]
