# Supplementary material for: The type of the functional cardiovascular response to upright posture is associated with arterial stiffness: a cross-sectional study in 470 volunteers
Source: BMC Cardiovasc Disord. 2016 May 23;16:101. doi: 10.1186/s12872-016-0281-8 (PMC4877753; doi:10.1186/s12872-016-0281-8)
Supplement: Additional file 5: — Table in pdf-format showing the analysis of explanatory factors for supine pulse wave velocity in multivariate analysis using continuous variables without categorization. (PDF 13 kb) [file 12872_2016_281_MOESM5_ESM.pdf]

Additional file 5. Analysis of explanatory factors for logarithmically transformed supine pulse wave velocity in multivariate analysis using continuous variables without categorization. Pulse wave velocity was logarithmically transformed for the analysis due to skewed distribution. The change in systemic vascular resistance and the change in cardiac index were analysed separately due to collinearity.

	Model 1: Constant 1.20767		Model 2: Constant 1.13718	
Variable	Coefficient	p-value	Coefficient	p-value
Change in systemic vascular resistance index during tilt	-	-	-0.00003	0.129
Change in cardiac index during tilt	0.08792	0.001	-	-
Body mass index (kg/m^2)	0.00732	0.005	0.00959	<0.001
Age (years)	0.00747	<0.001	0.00748	<0.001
Female sex	-0.01925	0.316	-0.03086	0.108
Systolic blood pressure at rest (mmHg)	0.00239	0.023	0.00221	0.029
Diastolic blood pressure at rest (mmHg)	0.00110	0.420	0.00124	0.362

The change in cardiac index and the change in systemic vascular resistance index were used as the main explanatory variables and in models 1 and 2, respectively. Both models have the same cofounders: BMI, age, sex and blood pressure. Lack of linearity in the data and collinearity between the changes in systemic vascular resistance index and cardiac index somewhat compromise this analysis.
