# Supplementary material for: The type of the functional cardiovascular response to upright posture is associated with arterial stiffness: a cross-sectional study in 470 volunteers
Source: BMC Cardiovasc Disord. 2016 May 23;16:101. doi: 10.1186/s12872-016-0281-8 (PMC4877753; doi:10.1186/s12872-016-0281-8)
Supplement: Additional file 6: — Table in pdf-format showing outcome of adjusted analyses of heart rate variability parameters in supine and upright positions using model 1 presented in Table 2. (PDF 13 kb) [file 12872_2016_281_MOESM6_ESM.pdf]

Additional file 6. Adjusted analyses of heart rate variability.

	Supine		Upright	
Variable	Coefficient	p-value	Coefficient	p-value
LF power				
Cluster: intermediate	0.119	0.718	-0.201	0.576
Cluster: sustainer	0.149	0.627	-0.405	0.205
HF power				
Cluster: intermediate	0.119	0.773	-0.365	0.384
Cluster: sustainer	0.057	0.884	-0.751	0.055
LF/HF ratio				
Cluster: intermediate	-0.106	0.761	0.076	0.841
Cluster: sustainer	-0.032	0.921	0.204	0.558

Outcome of adjusted analyses of heart rate variability parameters in supine and upright positions using model 1 presented in Table 3, adjusted with BMI-category, age-category, BP-category, and sex. Because there were no significant differences between clusters, only the coefficients and p-values for cluster membership are shown.

The male sex, constrictor cluster, lowest BMI tertile, lowest age tertile, and BP<135/85 mmHg in the laboratory served as reference categories. LF, low frequency; HF, high frequency.

The formula of the whole model would be: log(LF power) ~ Constant + cluster membership + BMI-category + AGE-category + BP category + sex + cluster membership*sex.
